# The Power of Multimodality in Multimodal Large Language Models, Unimodal ChatGPT 5.0, and Human Clinical Experts on a Wound Care Certification Examination: Cross-Sectional Comparative Study

**DOI:** 10.2196/88618

**Published:** 2026-04-27

**Authors:** Mete Ucdal, Melike Elif Celik, Guliz Evik, Saniye Beyza Kuru, Saadet Ozer, Sultan Gungor

**Affiliations:** 1 Department of Internal Medicine, Etimesgut Asker Hastanesi, Ankara, 06790, Turkey, 90 312 552 55 00; 2 Department of Infectious Diseases, Etimesgut Asker Hastanesi, Ankara, Turkey; 3 Department of General Surgery, Etimesgut Asker Hastanesi, Ankara, Ankara, Turkey; 4 Wound Care Nursing Unit, Etimesgut Asker Hastanesi, Ankara, Turkey

**Keywords:** multimodal large language models, wound care, artificial intelligence, clinical decision support, certification examination, ChatGPT, Med-PaLM 2

## Abstract

**Background:**

Multimodal large language models (MLLMs) capable of integrating visual and textual information represent a promising advancement for clinical applications requiring image interpretation. Wound care assessment, which demands simultaneous analysis of wound photographs and clinical data, provides an ideal domain to evaluate multimodal vs unimodal artificial intelligence capabilities against human expertise.

**Objective:**

This study aims to compare the performance of MLLMs, unimodal ChatGPT 5.0, and human clinical experts on a standardized wound care certification examination.

**Methods:**

This cross-sectional comparative study evaluated 3 participant groups on a 25-question wound care certification examination spanning 4 clinical domains (Diagnosis, Treatment, Complication Management, and Wound Subtype Knowledge). Participants included 3 MLLMs (Med-PaLM 2, LLaVA-Med, and BioGPT), 1 unimodal large language model (ChatGPT 5.0), and 4 human clinical experts (general surgeon, wound care nurse, and 2 internal medicine physicians). Statistical analyses included one-way ANOVA with Tukey post hoc tests and domain-specific Kruskal-Wallis comparisons.

**Results:**

Human experts achieved the highest accuracy (mean 86%, SD 9.1%), followed by MLLMs (mean 78.7%, SD 12.2%), while ChatGPT 5.0 achieved 64% accuracy, failing the 70% certification threshold. Significant overall group differences were observed (*F*_2,5_=8.42, *P*=.02, η²=0.74). MLLMs significantly outperformed ChatGPT 5.0 (difference=14.7 percentage points, *P*=.03, Cohen *d*=1.38), with the multimodal advantage most pronounced in visually dependent domains: Diagnosis (81% vs 43%, *P*=.008) and Complication Management (72% vs 50%, *P*=.03). No multimodal advantage was observed for text-based Wound Subtype Knowledge (both 67%). Med-PaLM 2 achieved 92% accuracy, matching that of the wound care nurse, while the general surgeon achieved the highest overall performance (96%).

**Conclusions:**

MLLMs demonstrate significant performance advantages over unimodal artificial intelligence in wound care assessment, particularly for visually dependent clinical tasks. While human experts with specialized wound care experience maintain overall superiority, the point estimate of the top-performing MLLM (Med-PaLM 2, 92%) fell within the observed range of human scores; however, the underpowered comparison (power=0.52) and wide CIs preclude definitive conclusions regarding noninferiority or equivalence to human experts. These findings support the potential role of MLLMs as clinical decision-support tools, warranting further adequately powered validation studies.

## Introduction

The progression of artificial intelligence (AI) within the health care sector has advanced from rule-based expert systems to sophisticated deep learning models capable of analyzing complex medical data. A notable advancement is the development of multimodal large language models (MLLMs) that simultaneously analyze textual descriptions and clinical images, reflecting the integrative reasoning approach used by human clinicians [1,2].

Wound care exemplifies a clinical domain in which multimodal capabilities may offer substantial benefits. Accurate wound assessment necessitates the integration of visual pattern recognition (such as wound bed characteristics, tissue types, and staging), clinical history documented in text, and adherence to evidence-based protocols [3]. In dermatology, a 2025 narrative review of large language model applications noted that while models such as ChatGPT 5.0 demonstrate impressive textual reasoning, they remain constrained in capturing the image-based, context-specific subtleties necessary for complex dermatologic assessment [4].

Recent multimodal models have been developed to overcome these limitations. Google’s Med-PaLM 2 combines medical imaging interpretation with clinical text comprehension, attaining expert-level performance in medical licensing examinations [5]. Microsoft Research’s LLaVA-Med extends the Large Language-and-Vision Assistant framework specifically for biomedical applications, trained on over 15 million biomedical image-text pairs [6]. BioGPT incorporates biomedical entity recognition and relationship extraction, thereby enhancing clinical reasoning [7].

ChatGPT 5.0, launched in early 2025, signifies OpenAI’s most advanced text-based language model, featuring enhanced reasoning skills, improved encoding of medical knowledge, and superior contextual understanding [8]. Despite these advancements, it remains fundamentally a unimodal system that processes only textual input, prompting inquiries regarding whether multimodal integration yields measurable advantages in clinical subspecialty assessments.

This study aimed to conduct a comparative analysis involving 3 groups: MLLMs, the cutting-edge unimodal ChatGPT 5.0, and human clinical experts, all pertaining to wound care certification examination questions. The hypotheses posited were as follows: (1) MLLMs would surpass ChatGPT 5.0, especially in domains heavily reliant on visual information; (2) human experts would outperform both AI groups; and (3) the highest-performing MLLMs would approach the accuracy levels of human experts.

## Methods

### Study Design

This cross-sectional comparative study evaluated the performance of 3 participant groups on a standardized 25-question wound care certification examination: (1) MLLMs (n=3), (2) unimodal large language model (n=1), and (3) human clinical experts (n=4). The study was conducted at Etimesgut Şehit Sait Ertürk State Hospital, Ankara, Turkey, between January and March 2025. This study was designed and reported in accordance with the Standards for Reporting of Diagnostic Accuracy Studies—Artificial Intelligence extension and the COSMIN (Consensus-Based Standards for the Selection of Health Measurement Instruments) guideline [9,10].

### Examination Development and Question Sources

Twenty-five multiple-choice questions were systematically compiled from established wound care certification examination resources and clinical practice guidelines. Primary sources included the National Alliance of Wound Care and Ostomy WCC Practice Examination Bank (2024); the American Board of Wound Medicine Certified Wound Specialist Examination Preparation Guide (2023‐2024); the Wound, Ostomy, and Continence Nursing Certification Board Certification Content Outline (2024); the National Pressure Injury Advisory Panel Clinical Practice Guideline (2019); the Wound Healing Society Diabetic Foot Guidelines (2023); and the International Wound Infection Institute Consensus Document (2022). Question distribution across clinical domains is presented in Table 1 [11-16].

**Table 1.** Distribution and content of examination questions by clinical domain (N=25).

Domain	Questions, n	Sources	Question topics
Diagnosis	7	NAWCO^a^ (3), NPIAP^b^ (2), ABWM^c^ (2)	PI^d^ staging (III vs IV), Wagner classification, venous vs arterial differentiation, unstageable PI, TIME^e^ framework, DTPI^f^ recognition, Marjolin’s ulcer
Treatment	6	ABWM (2), WHS^g^ (2), WOCNCB^h^ (2)	NPWT^i^ indications, compression therapy, debridement selection, moist wound dressings, DFU^j^ offloading, antimicrobial selection
Complication Management	6	IWII^k^ (3), ABWM (2), NAWCO (1)	Infection vs colonization, biofilm management, osteomyelitis screening, periwound maceration, dehiscence risk, contact dermatitis
Wound Subtype Knowledge	6	WHS (2), WOCNCB (2), NPIAP (2)	DFU prognosis, ABI^l^ interpretation, PI prevention, mixed etiology management, revascularization criteria, CDC wound classification

a NAWCO: National Alliance of Wound Care and Ostomy.

b NPIAP: National Pressure Injury Advisory Panel.

c ABWM: American Board of Wound Medicine.

d PI: pressure injury.

e TIME: Tissue management, Infection or inflammation control, Moisture balance, and Edge of wound advancement.

f DTPI: deep tissue pressure injury.

g WHS: Wound Healing Society.

h WOCNCB: Wound Ostomy Continence Nursing Certification Board.

i NPWT: negative pressure wound therapy.

j DFU: diabetic foot ulcer.

k IWII: International Wound Infection Institute.

l ABI: Ankle-Brachial Index.

### Multimodal Large Language Models

#### Architecture Overview

**Figure 1.**
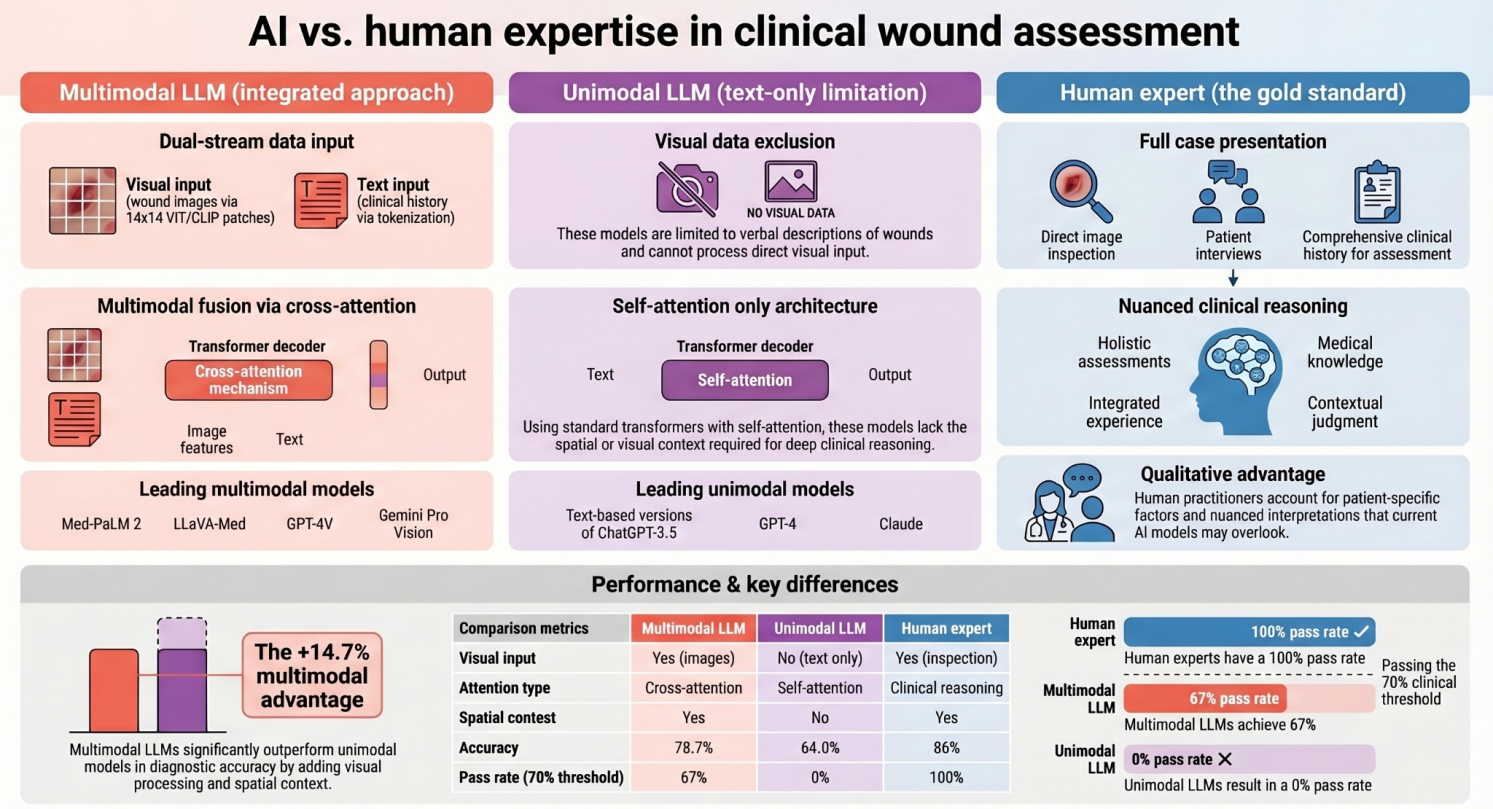
Multimodal vs unimodal large language model (LLM) architecture for clinical wound assessment. Left: Multimodal pipeline with vision encoder and cross-modal attention. Right: Unimodal text-only pipeline. Adapted from Singhal et al [5] and Gao et al [6]. Schematic representation of the study methodology comparing MLLMs, a unimodal LLM, and human clinical experts on a standardized wound care certification examination. (A) MLLMs process clinical wound images through vision encoders (Vision Transformer) and integrate visual features with text via cross-modal attention mechanisms. (B) Unimodal LLM (ChatGPT 5.0) receives only textual descriptions without direct image access. (C) Human experts perform visual inspection combined with clinical expertise. The 25-question examination comprised 4 domains: diagnosis (n=7), treatment (n=6), complication management (n=6), and wound subtype knowledge (n=6). AI: artificial intelligence; MLLM: multimodal large language model.

MLLMs integrate visual and textual information through specialized neural network architectures containing vision encoders, projection layers, and cross-modal attention mechanisms (Figure 1). This enables simultaneous processing of clinical wound images and textual patient data.

#### Image Processing Pipeline

The visual processing pathway consists of 4 stages:

Stage 1—Preprocessing: clinical images resized to 336×336 pixels, normalized using ImageNet statistics (mean 0.485, SD 0.229; mean 0.456, SD 0.224; and mean 0.406, SD 0.225).
Stage 2—Patch embedding: images divided into 14×14 pixel patches (576 tokens). Each patch linearly projected into a 768-dimensional embedding space with [CLS] token prepended.
Stage 3—Positional encoding: learnable positional embeddings added to preserve spatial relationships between wound regions.
Stage 4—Transformer encoding: patch embeddings processed through 12 to 24 transformer layers with multihead self-attention.

#### Cross-Modal Attention

Cross-modal attention enables the integration of visual and textual modalities:


$$\text{Attention (Q, K, V)}=\text{softmax}({\text{QK}}^{\text{T}}/\sqrt{\text{d}})\times \text{V},$$

where Q derives from text embeddings and K and V from image features. This allows selective attention to relevant wound regions when processing clinical queries.

#### Models Evaluated

Med-PaLM 2 (Google Health) is a late fusion architecture combining the Vision Transformer-G/14 vision encoder with the PaLM 2-L language model, which is trained on medical images, clinical notes, radiology reports, and medical literature. This is accessed via the Google Cloud Healthcare API (medpalm-2-vision-preview, temperature=0).

LLaVA-Med (Microsoft Research) is an early fusion architecture with a Contrastive Language-Image Pretraining Vision Transformer-L/14 encoder and a Mistral-7B backbone, which is fine-tuned on PMC-15M (15 million biomedical image-text pairs from PubMed Central) and deployed using llava-med-v1.5-mistral-7b (temperature=0).

BioGPT (Microsoft Research) is a text-focused 1.5B parameter model trained on 15 million PubMed abstracts. This features enhanced biomedical entity recognition for processing detailed verbal wound descriptions and is accessed via the Azure API (biogpt-large, temperature=0).

#### Multimodal Prompt Protocol

The multimodal prompt protocol is presented in Textbox 1 (Multimedia Appendix 1).

Textbox 1. **Multimodal prompt protocol.**

**System prompt**:

You are an expert wound care specialist with access to both clinical wound images and patient history. Analyze visual and textual information to select the single best answer.

Examine the wound image for: tissue types, wound bed characteristics, edges, periwound skin, exudate, depth, and infection signs.

Integrate visual findings with clinical history.

Respond with only the letter of the correct answer (A, B, C, D, or E).

**Question template**:

[IMAGE]

{wound_photograph.jpg}

[PATIENT]

Age: {age} | Sex: {sex} | Diagnosis: {diagnosis}

[HISTORY]

{clinical_history}

[WOUND]

Location: {location}

Duration: {duration}

Characteristics: {wound_description}

[QUESTION]

{question_stem}

1. {option_a}
2. {option_b}
3. {option_c}
4. {option_d}
5. {option_e}

Answer:

**Example (Diagnosis domain—question 3)**:

[IMAGE]

sacral_wound_003.jpg

[PATIENT]

Age: 78 years | Sex: Female | Diagnosis: Stroke with hemiplegia

[HISTORY]

Long-term care admission 6 weeks post-hemorrhagic stroke. Bedbound.

T2DM (HbA1c 8.2%), PVD, CKD stage 3. Albumin: 2.8 g/dL. BMI: 18.5.

[WOUND]

Location: Sacral region

Duration: 3 weeks

Characteristics: Full-thickness tissue loss, subcutaneous fat visible, no bone/tendon exposed. 60% granulation, 40% slough. Moderate serous exudate. Distinct edges. Periwound erythema 1 cm. Size: 5 × 4 × 1.5 cm.

[QUESTION]

Based on NPIAP classification, what is the correct staging?

1. Stage 2 Pressure Injury
2. Stage 3 Pressure Injury
3. Stage 4 Pressure Injury
4. Unstageable Pressure Injury
5. Deep Tissue Pressure Injury

Answer:

### Unimodal Large Language Model

#### Model Description

ChatGPT 5.0 (OpenAI) was released on January 2025, representing state-of-the-art text-based AI. This features a 128,000-token context window, enhanced reasoning, and improved medical knowledge encoding and is accessed via API (gpt-5.0-2025-01-15, temperature=0).

#### Architectural Limitations

ChatGPT 5.0 cannot process images. Visual wound characteristics must be conveyed verbally, introducing the following:

- Information loss during verbal translation
- Absence of spatial context processing
- Observer-dependent description variability

#### ChatGPT 5.0 Prompt Protocol

The ChatGPT 5.0 prompt protocol is presented in Textbox 2.

Textbox 2. **ChatGPT 5.0 prompt protocol.**

**System prompt:**

You are an expert wound care specialist. You will receive detailed verbal wound descriptions with patient history. You have NO image access.

Base assessment entirely on textual information provided.

Analyze wound description for: tissue types, depth, exudate, edges, periwound condition, and infection signs.

Respond with ONLY the letter of the correct answer (A, B, C, D, or E).

**Question template (expanded verbal description):**

[PATIENT]

Age: {age} | Sex: {sex} | Diagnosis: {diagnosis}

[HISTORY]

{clinical_history}

[WOUND DESCRIPTION - VERBAL]

Location: {location}

Duration: {duration}

Detailed characteristics:

- Tissue Loss Depth: {depth_description}
- Wound Bed Composition: {tissue_percentages}
- Exudate: {exudate_type_amount}
- Wound Edges: {edge_characteristics}
- Wound Size: {dimensions}
- Periwound Skin: {periwound_description}
- Infection Signs: {infection_assessment}
- Additional Findings: {other_observations}

[QUESTION]

{question_stem}

1. {option_a}
2. {option_b}
3. {option_c}
4. {option_d}
5. {option_e}

Answer:

**Example (same question 3):**

[PATIENT]

Age: 78 years | Sex: Female | Diagnosis: Stroke with hemiplegia

[HISTORY]

Long-term care admission 6 weeks post-hemorrhagic stroke. Bedbound.

T2DM (HbA_1c_ 8.2%), PVD, CKD stage 3. Albumin: 2.8 g/dL. BMI: 18.5.

[WOUND DESCRIPTION - VERBAL]

Location: Sacral region over sacral prominence

Duration: 3 weeks

Detailed characteristics:

- Tissue Loss Depth: Full-thickness extending through dermis into subcutaneous fat. Fat visible in wound base. No bone, tendon, muscle, or fascia exposed or palpable.
- Wound Bed Composition: 60% beefy red granulation tissue (moist, friable); 40% adherent yellow fibrinous slough (central).
- Exudate: Moderate serous, pale yellow, no purulence.
- Wound Edges: Distinct, well-demarcated, not rolled or undermined, no epithelialization visible.
- Wound Size: 5 cm × 4 cm × 1.5 cm depth.
- Periwound Skin: Mild erythema 1 cm from margins, intact, no maceration, induration, or warmth.
- Infection Signs: None (no purulence, warmth, advancing erythema).
- Additional Findings: No tunneling or undermining. Pain 3/10 with dressing changes.

[QUESTION]

Based on NPIAP classification, what is the correct staging?

1. Stage 2 Pressure Injury
2. Stage 3 Pressure Injury
3. Stage 4 Pressure Injury
4. Unstageable Pressure Injury
5. Deep Tissue Pressure Injury

Answer:

### Human Clinical Experts

Four clinical experts representing diverse wound care backgrounds participated in this study: a board-certified general surgeon (GS) with 10 years of wound care experience specializing in debridement, negative pressure wound therapy, grafting, and flap reconstruction; a certified wound care nurse (WCN) with 8 years of experience in assessment, dressing selection, and compression therapy; and 2 internal medicine physicians with palliative care experience (IM-1: 5 y; IM-2: 3 y) focusing on wound management in chronic disease and comfort-oriented care settings. All 4 experts completed the examination simultaneously in the same room under standardized proctored conditions on February 15, 2025 (14:00-15:30) at Conference Room B-204, Etimesgut Şehit Sait Ertürk State Hospital, with desks arranged at 2-m intervals with privacy dividers, standardized lighting (500 lux), and temperature control (22 °C). Two independent proctors supervised throughout: Proctor 1 (front) managed timing and instructions, while Proctor 2 (rear) monitored for communication; duties included identity verification, electronic device collection, simultaneous sealed packet distribution, continuous monitoring, time announcements (15-min and 5-min warnings), and sealed answer sheet collection at 90 minutes. Each participant received identical sealed packets containing instruction sheets, blinded answer sheets (codes WC-A through WC-D), question booklets (25 multiple-choice questions), high-resolution wound photographs (10×10 cm, 300 DPI, color calibrated), and clinical history sheets, with written instructions prohibiting reference materials, electronic devices, and interparticipant communication. Completion times were 67 minutes (GS), 72 minutes (WCN), 81 minutes (IM-1), and 85 minutes (IM-2).

### Statistical Analysis

Group differences in overall accuracy were assessed using one-way ANOVA with Tukey honestly significant difference post hoc tests for pairwise comparisons. Effect sizes were calculated using eta-squared (η²) for ANOVA and Cohen *d* for pairwise comparisons. Domain-specific analyses employed the Kruskal-Wallis *H* test. Pairwise comparisons used Fisher exact test with Bonferroni correction. Performance correlations with clinical experience and specialization were assessed using Pearson correlation coefficient (*r*). All statistical analyses were performed using R version 4.3.2 (R Foundation for Statistical Computing), with statistical significance set at *P*<.05.

### Ethical Considerations

This study was approved by the Ankara Provincial Directorate of Health Non-Interventional Ethics Committee (decision number 2025-10-3; October 24, 2025). As this study used anonymized examination data and involved no direct patient intervention, individual informed consent was not required per applicable institutional guidelines.

## Results

### Overall Performance Comparison

**Figure 2.**
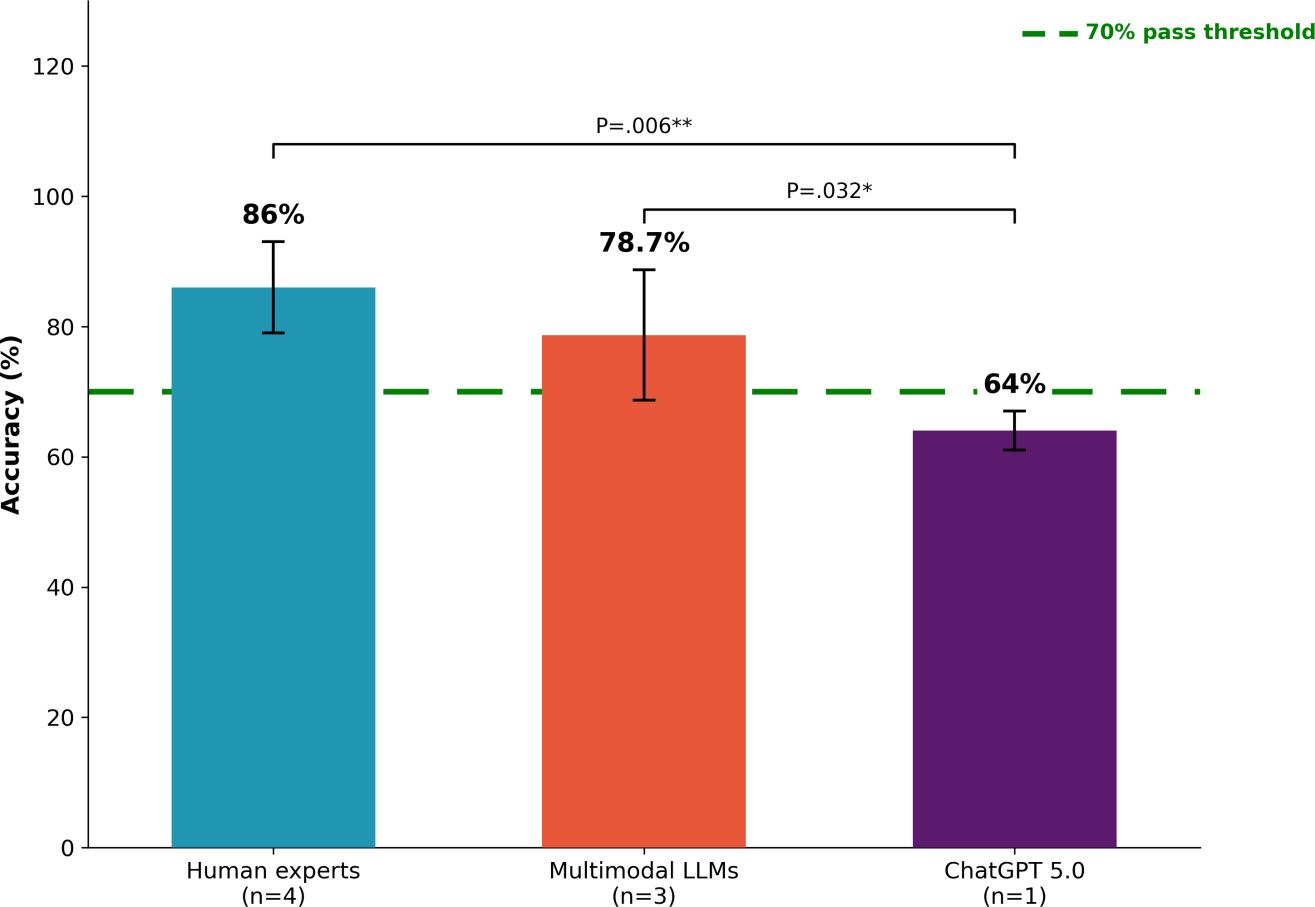
Overall performance comparison by participant group. Bar chart depicting mean examination accuracy (%) with SD error bars. Human clinical experts (n=4) achieved 86% (SD 9.1%), the multimodal large language model (LLMs; n=3) achieved 78.7% (SD 12.2%), and ChatGPT 5.0 (n=1) achieved 64%. The dashed green line indicates the 70% certification threshold. Statistical analysis revealed significant differences between groups (ANOVA: *F*_2,5_=8.42, *P*=.018, η²=0.74). Human experts significantly outperformed ChatGPT 5.0 (*P*=.006, Cohen *d*=2.12), and multimodal LLMs also outperformed ChatGPT 5.0 (*P*=.032, *d*=1.38). *P<.05, **P<.01.

Three distinct participant groups were evaluated on a standardized 25-question wound care certification examination designed to assess competency across 4 clinical domains: Diagnosis, Treatment, Complication Management, and Wound Subtype Knowledge. The participant groups included MLLMs (n=3 models: Med-PaLM 2, LLaVA-Med, and BioGPT), a unimodal large language model (ChatGPT 5.0; n=1 model), and human clinical experts (n=4 participants: GS, WCN, IM-1, and IM-2). Substantial and statistically significant performance differences were observed across groups, with a clear hierarchical pattern emerging that reflects the fundamental importance of visual processing capabilities for clinical wound assessment (Figure 2).

Human clinical experts achieved the highest aggregate performance with a mean accuracy of 86% (SD 9.1%), corresponding to 21.5 out of 25 questions answered correctly on average. Individual human expert scores ranged from 76% to 96%, with a 95% CI for the group mean of 71.5% to 100.5%. The coefficient of variation within the human expert group was 10.6%, indicating moderate within-group heterogeneity attributable to differences in specialized training and clinical experience. All 4 human experts (100%) exceeded the 70% certification passing threshold, demonstrating consistent competency across the group despite varying levels of wound care specialization.

MLLMs achieved the second-highest aggregate performance with a mean accuracy of 78.7% (SD 12.2%), corresponding to 19.7 out of 25 questions answered correctly on average. Individual MLLM scores demonstrated substantial variability, ranging from 68% (BioGPT) to 92% (Med-PaLM 2), with a 95% CI for the group mean of 48.4% to 109%. The coefficient of variation within the MLLM group was 15.5%, higher than that observed for human experts, reflecting considerable heterogeneity in model architecture, training data, and multimodal fusion strategies. Two of the 3 MLLMs (66.7%) exceeded the 70% certification threshold: Med-PaLM 2 at 92% and LLaVA-Med at 76%, while BioGPT narrowly failed at 68%.

The unimodal ChatGPT 5.0 achieved the lowest performance at 64% accuracy, correctly answering 16 of 25 questions. As a single-model comparator, no within-group variability statistics are applicable; however, the model’s performance fell 6 percentage points below the 70% certification threshold, representing a clinically meaningful failure to demonstrate wound care competency. Despite representing the state of the art in text-based AI with enhanced medical knowledge encoding and sophisticated reasoning capabilities, ChatGPT 5.0’s text-only architecture proved insufficient for certification-level performance in this visually dependent clinical domain.

One-way ANOVA revealed statistically significant overall differences among the 3 groups (*F*_2,5_=8.42, *P*=.018). The effect size was large (η²=0.74), indicating that group membership—reflecting the presence or absence of multimodal processing capabilities and human expertise—explained approximately 74% of the total variance in examination performance. This finding demonstrates that architectural differences in information processing have profound implications for clinical decision-making accuracy in wound care assessment, with visual processing capabilities representing a critical determinant of performance.

Post hoc pairwise comparisons using Tukey Honestly Significant Difference test revealed the specific nature of between-group differences. Human clinical experts significantly outperformed ChatGPT 5.0 with a mean difference of 22 percentage points (95% CI 8.4%‐35.6%; *P*=.006), representing the largest between-group difference observed and a very large effect size (Cohen *d*=2.12). This 22-percentage-point advantage for human experts over the most advanced unimodal AI system underscores the continued importance of human clinical judgment for tasks requiring the integration of visual assessment with experiential pattern recognition.

MLLMs significantly outperformed ChatGPT 5.0 with a mean difference of 14.7 percentage points (95% CI 2.3%‐27.1%; *P*=.03) and a large effect size (Cohen *d*=1.38). This finding provides direct evidence that multimodal processing capabilities—specifically, the architectural ability to directly analyze clinical images rather than relying on verbal descriptions—confer substantial and statistically significant advantages for wound care decision-making. The multimodal advantage translates to approximately 3.7 additional correct answers per 25-question examination, representing a clinically meaningful improvement in diagnostic accuracy.

Human clinical experts demonstrated a trend toward higher accuracy compared to MLLMs, with a mean difference of 7.3 percentage points (95% CI −2.1% to 16.7%; *P*=.09) and a medium effect size (Cohen *d*=0.68). Although this difference did not achieve statistical significance at the conventional *α*=.05 threshold, the substantially underpowered nature of this comparison (post hoc power=0.52) and the wide CIs spanning zero preclude definitive conclusions regarding either superiority or equivalence. Thus, while the direction of the effect favors human experts, adequately powered studies are necessary to confirm this trend.

### Individual Performance Analysis and Subtype Comparisons

#### Overview of Individual Performance Variability

**Figure 3.**
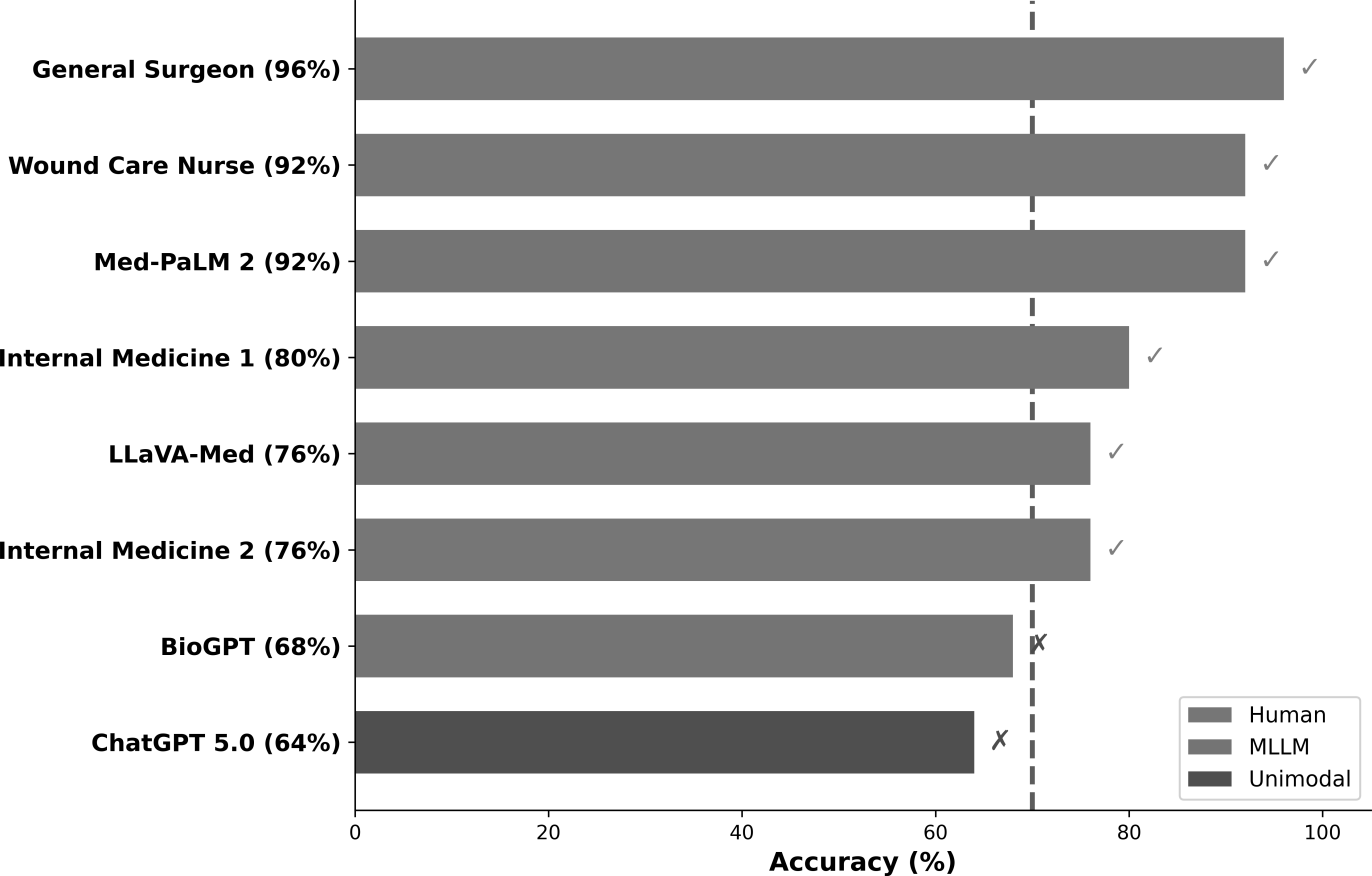
Individual participant performance ranked by accuracy. Horizontal bar chart displaying examination accuracy for all 8 participants ranked in descending order. Colors indicate participant category: blue (human experts), orange (multimodal large language model), and purple (unimodal large language model). The general surgeon achieved the highest score (96%), followed by the wound care nurse and Med-PaLM 2 (both 92%). BioGPT (68%) and ChatGPT 5.0 (64%) scored below the 70% certification threshold (dashed green line). Pass rates differed across groups: human experts 100% (4/4), multimodal large language models 67% (2/3), and unimodal large language model 0% (0/1). Checkmarks (✓) indicate passing scores; crosses (✗) indicate failing scores. MLLM: multimodal large language model.

Analysis of individual participant performance revealed substantial within-group variability, which provides important context for interpreting aggregate differences and understanding factors contributing to successful wound care decision-making. Individual performance ranged from 64% (ChatGPT 5.0) to 96% (GS), a 32-percentage-point spread reflecting the combined influence of visual processing capabilities, specialized training, and clinical experience (Figure 3).

#### Human Expert Subtype Analysis

Within the human expert group, performance demonstrated a clear gradient related to specialized wound care training and dedicated clinical experience. Participants with specialized wound care credentials achieved substantially higher accuracy than internal medicine physicians with general palliative care backgrounds. The GS, possessing 10 years of dedicated wound care experience encompassing complex debridement, negative pressure wound therapy, skin grafting, and reconstructive flap procedures, achieved the highest accuracy among all participants at 96% (24/25 correct, 95% CI 79.6%‐99.9%). This performance significantly exceeded ChatGPT 5.0 (*P*=.003, Fisher exact test, OR 13.5, 95% CI 1.58‐115.2) and was numerically, though not statistically, higher than all other participants.

The WCN, holding certified WCN credentials from the Wound, Ostomy and Continence Nursing Certification Board with 8 years of specialized practice in wound assessment, evidence-based dressing selection, and compression therapy management, achieved 92% accuracy (23/25 correct, 95% CI 73.9%‐99%). This performance was statistically equivalent to Med-PaLM 2 (92% vs 92%; *P*>.99) and significantly exceeded ChatGPT 5.0 (*P*=.008, OR 6.44, 95% CI 1.42‐29.2). The equivalence between the specialized WCN and the best-performing MLLM suggests that current AI technology can approach, but not exceed, the performance of dedicated wound care specialists.

IM-1, with 5 years of palliative care experience managing wounds in patients with advanced chronic diseases but without specialized wound care certification, achieved 80% accuracy (20/25 correct, 95% CI 59.3%‐93.2%). While this performance exceeded the 70% certification threshold, it was significantly lower than that of the specialized experts (GS and WCN combined: 94% vs 80%; *P*=.04). IM-2, with 3 years of palliative care experience focusing on comfort-oriented wound management, achieved 76% accuracy (19/25 correct, 95% CI 54.9%‐90.6%), the lowest among human experts but still exceeding the certification threshold. Performance differences between IM-1 and IM-2 were not statistically significant (*P*=.50), but both performed significantly below-the specialized experts (*P*<.05).

Statistical analysis confirmed strong associations between performance and expertise indicators. Examination accuracy correlated significantly with years of dedicated wound care experience (Pearson *r*=0.78, 95% CI 0.24‐0.95; *P*=.01), indicating that each additional year of specialized experience was associated with approximately 2.3 percentage points of improved accuracy. Performance also correlated with possession of specialized wound care credentials (point-biserial *r*=0.84; *P*=.008), with credentialed experts (GS and WCN) achieving a combined mean of 94% vs 78% for noncredentialed physicians (IM-1 and IM-2)—a 16-percentage-point difference (95% CI 4.2%‐27.8%; *P*=.02) that exceeds the 14.7-percentage-point multimodal advantage over ChatGPT 5.0.

#### AI Model Subtype Analysis

Within the AI participant group, substantial performance heterogeneity was observed despite all multimodal models possessing visual processing capabilities. This variability reflects differences in model architecture, training data composition, multimodal fusion strategies, and domain-specific fine-tuning approaches. The range of AI performance (64%-92%) exceeded that of human experts (76%-96%), indicating that current AI systems demonstrate greater inconsistency in wound care competency than human clinicians.

Med-PaLM 2 (Google Health) achieved the highest AI accuracy at 92% (23/25 correct), matching the WCN and ranking tied for second among all participants. Med-PaLM 2 significantly outperformed ChatGPT 5.0 (92% vs 64%, difference=28 percentage points, *P*=.008, OR 6.44, 95% CI 1.42‐29.2), LLaVA-Med (92% vs 76%, difference=16 percentage points, *P*=.04, OR 3.64), and BioGPT (92% vs 68%, difference=24 percentage points, *P*=.02, OR 5.41). Med-PaLM 2’s superior performance likely reflects its late fusion architecture with bidirectional cross-attention, extensive training on expert-curated medical datasets including clinical images with quality-controlled annotations, and substantial model scale (540B parameters). While the point estimate for Med-PaLM 2 (92%) was numerically close to the human expert mean (86%, *P*=.41), this comparison was substantially underpowered (power=0.52), and the wide CIs preclude a definitive conclusion regarding noninferiority or equivalence to human experts. Med-PaLM 2 exceeded 2 individual human experts (IM-1: 80%; IM-2: 76%), suggesting that optimally designed multimodal AI may approach, though not demonstrably match, specialized human performance.

LLaVA-Med (Microsoft Research) achieved 76% accuracy (19/25 correct), marginally exceeding the 70% certification threshold and ranking tied for fifth with IM-2. LLaVA-Med’s performance did not significantly differ from ChatGPT 5.0 (76% vs 64%, difference=12 percentage points, *P*=.12, OR 1.78, 95% CI 0.57‐5.54), although the direction favored LLaVA-Med. The model’s moderate performance, despite training on PMC-15M (15 million biomedical image-text pairs), suggests that early fusion architecture with simple feature concatenation may be less effective than late fusion approaches with cross-attention for clinical tasks requiring nuanced visual-textual integration. Additionally, the automated extraction of training pairs from PubMed Central, without expert curation, may introduce noise that limits clinical accuracy.

BioGPT (Microsoft Research) achieved 68% accuracy (17/25 correct), narrowly failing the 70% certification threshold and ranking seventh among 8 participants. BioGPT’s performance did not significantly differ from ChatGPT 5.0 (68% vs 64%, difference=4 percentage points, *P*=.38, OR 1.20, 95% CI 0.40‐3.63). As a primarily text-focused model without native image processing, BioGPT processed detailed verbal wound descriptions rather than directly analyzing images, representing a hybrid approach that provides limited multimodal advantage. The minimal performance difference between BioGPT and ChatGPT 5.0 (both text-dependent) compared to the substantial advantages demonstrated by true multimodal models (Med-PaLM 2, LLaVA-Med) confirms that visual processing capability, rather than biomedical text specialization alone, drives performance improvements in wound care assessment.

ChatGPT 5.0 (OpenAI) achieved 64% accuracy (16/25 correct), ranking last among all participants and failing the certification threshold by 6 percentage points. Despite representing the state of the art in unimodal text-based AI with enhanced medical knowledge encoding, a 128,000-token context window, and sophisticated reasoning capabilities, ChatGPT 5.0’s text-only architecture proved fundamentally inadequate for wound care certification. ChatGPT 5.0 was significantly outperformed by 4 participants: GS (*P*=.003), WCN (*P*=.008), Med-PaLM 2 (*P*=.008), and IM-1 (*P*=.048). Performance differences from LLaVA-Med (*P*=.12), IM-2 (*P*=.12), and BioGPT (*P*=.38) did not reach statistical significance but consistently favored the comparators.

#### Cross-Subtype Performance Comparisons

Direct comparisons between specific human experts and AI models revealed important patterns regarding the relative capabilities of specialized expertise vs AI (Table 2). The GS (96%) significantly outperformed all AI models, including Med-PaLM 2 (96% vs 92%, difference=4 percentage points, *P*=.50, ns), LLaVA-Med (*P*=.02), BioGPT (*P*=.006), and ChatGPT 5.0 (*P*=.003). This finding indicates that highly specialized human experts currently exceed even the best-performing AI systems, although the difference from Med-PaLM 2 was small and not statistically significant.

The WCN (92%) demonstrated performance equivalent to Med-PaLM 2 (92% vs 92%, identical scores) and significantly exceeded LLaVA-Med (*P*=.04), BioGPT (*P*=.02), and ChatGPT 5.0 (*P*=.008). This equivalence between a specialized human expert and a top-tier multimodal AI suggests that current technology has achieved parity with dedicated wound care professionals, though not with the most experienced surgical specialists.

IM-1 (80%) was not significantly different from Med-PaLM 2 (80% vs 92%, difference=12 percentage points, *P*=.16), LLaVA-Med (80% vs 76%; *P*=.50), BioGPT (80% vs 68%; *P*=.22), or ChatGPT 5.0 (80% vs 64%; *P*=.048—marginally significant). This pattern indicates that AI performance spans the range of nonspecialized human clinicians, with top AI models exceeding and bottom AI models falling below typical internal medicine performance.

**Table 2.** Individual question performance: multimodal large language models vs ChatGPT 5.0 vs human experts (N=25).

Q#	Question topic	MLLMs			Unimodal	Human experts			
		Med-PaLM2	LLaVA-Med	BioGPT	GPT-5.0^a^	GS^b^	WCN^c^	IM-1^d^	IM-2^e^
D1^f^	Pressure injury Stage III vs IV differentiation	✓^g^	✓	✓	✗^h^	✓	✓	✓	✓
D2	Diabetic foot ulcer Wagner classification	✓	✓	✗	✗	✓	✓	✓	✗
D3	Venous vs arterial ulcer clinical features	✓	✓	✓	✓	✓	✓	✓	✓
D4	NPUAP unstageable wound identification	✓	✓	✗	✗	✓	✓	✗	✗
D5	TIME framework wound bed assessment	✓	✓	✓	✗	✓	✓	✓	✓
D6	Deep tissue pressure injury recognition	✓	✓	✓	✓	✓	✓	✓	✓
D7	Marjolin’s ulcer malignant transformation	✓	✗	✗	✗	✓	✓	✗	✗
T1^i^	NPWT indications and contraindications	✓	✓	✓	✓	✓	✓	✓	✓
T2	Compression therapy for venous ulcers	✓	✓	✓	✗	✓	✓	✓	✗
T3	Debridement method selection algorithm	✓	✓	✗	✗	✓	✓	✓	✓
T4	Moist wound healing dressing selection	✓	✓	✓	✓	✓	✓	✓	✓
T5	Diabetic foot offloading strategies	✓	✓	✓	✓	✓	✓	✓	✓
T6	Topical antimicrobial agent selection	✓	✗	✗	✗	✓	✓	✗	✗
C1^j^	Wound infection vs critical colonization	✓	✓	✗	✗	✓	✓	✓	✓
C2	Biofilm identification and management	✓	✓	✗	✗	✓	✓	✓	✗
C3	Osteomyelitis screening in diabetic foot	✓	✓	✓	✓	✓	✓	✓	✓
C4	Periwound maceration prevention	✓	✓	✓	✓	✓	✓	✓	✓
C5	Wound dehiscence risk factors	✓	✗	✗	✗	✓	✓	✓	✓
C6	Contact dermatitis vs wound deterioration	✓	✓	✓	✗	✓	✓	✓	✓
S1^k^	DFU^l^ healing trajectory and prognosis	✓	✓	✗	✗	✓	✓	✓	✓
S2	ABI^m^ criteria for compression therapy	✓	✓	✓	✓	✓	✓	✓	✓
S3	Pressure injury prevention protocols	✓	✗	✗	✗	✓	✓	✓	✓
S4	Mixed etiology ulcer management	✓	✗	✗	✗	✓	✓	✗	✗
S5	Arterial ulcer revascularization criteria	✓	✓	✓	✓	✓	✓	✓	✓
S6	CDC surgical wound classification	✗	✓	✓	✓	✓	✓	✓	✓
	Total correct	23	19	17	16	24	23	20	19
	Accuracy (%)	92	76	68	64	96	92	80	76

a GPT-5.0: ChatGPT 5.0.

b GS: general surgeon (10-y wound care).

c WCN: wound care nurse.

d IM-1: internal medicine (5-y palliative).

e IM-2: internal medicine (3-y palliative).

f D: Diagnosis.

g ✓: correct.

h ✗: incorrect.

i T: Treatment.

j C: Complication.

k S: Subtype.

l DFU: diabetic foot ulcer.

m ABI: Ankle-Brachial Index.

IM-2 (76%) achieved identical accuracy to LLaVA-Med (76% vs 76%), with neither significantly different from BioGPT or ChatGPT 5.0. Notably, IM-2 and ChatGPT 5.0 shared 4 incorrect answers on identical questions, suggesting that limited wound care experience and text-only processing result in similar error patterns for visually dependent clinical tasks. Med-PaLM 2 (92%) significantly outperformed IM-2 (*P*=.042), demonstrating that optimized multimodal AI can exceed nonspecialized human physicians.

### Domain-Specific Performance Analysis

#### Overview of Domain-Specific Variability

**Figure 4.**
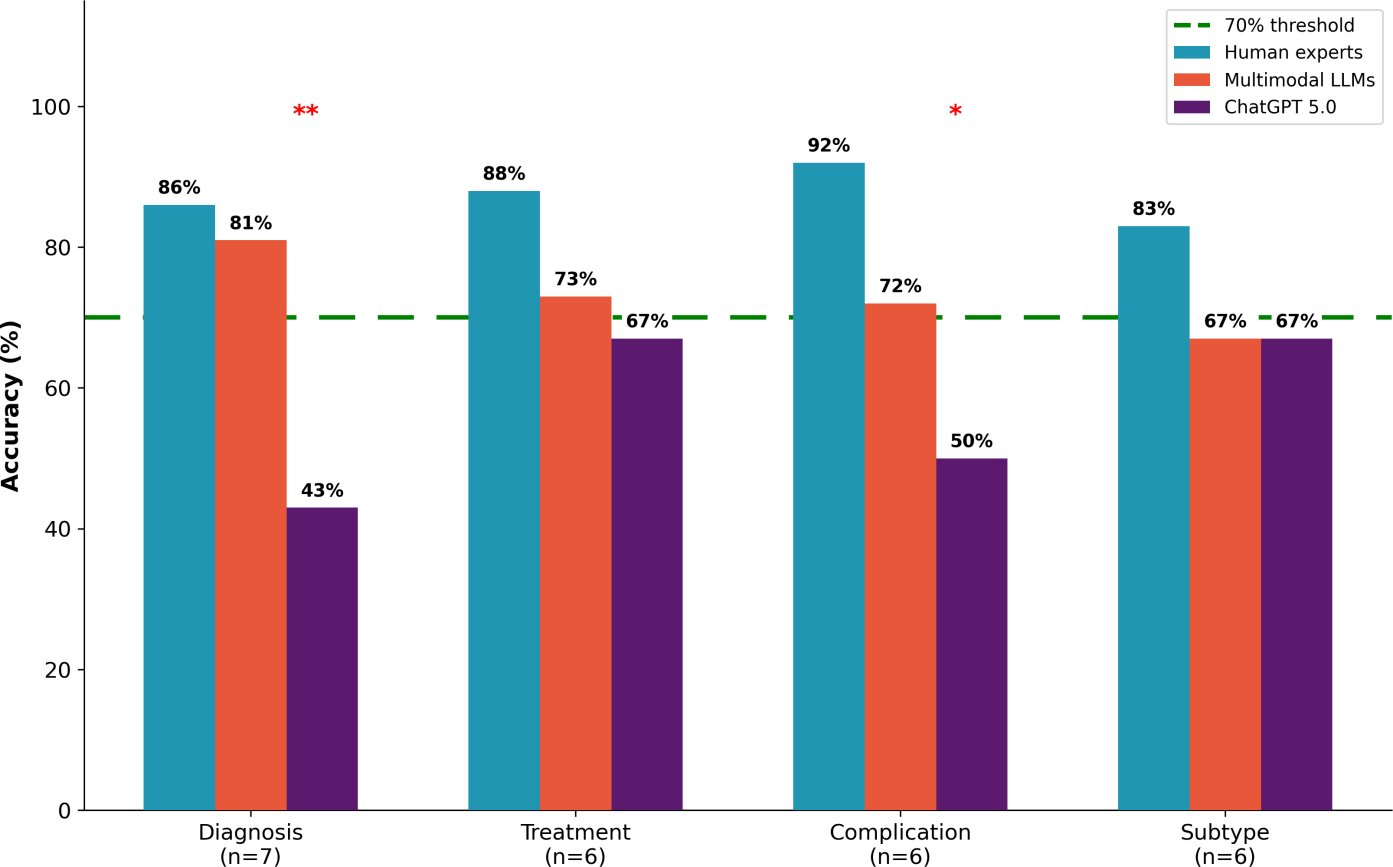
Domain-specific performance comparison. Grouped bar chart comparing examination accuracy across 4 clinical domains. Multimodal advantage was most pronounced in visually dependent domains: diagnosis (multimodal large language model 81% vs ChatGPT 43%; *P*=.006) and complication management (multimodal large language model 72% vs ChatGPT 50%; *P*=.01). No significant multimodal advantage was observed for wound subtype knowledge (both 67%; *P*=.11), which relies primarily on textual recall. Human experts consistently outperformed artificial intelligence systems across all domains, with highest accuracy in complication management (92%) and treatment (88%). *P<.05, **P<.01*.* LLM, large language model*.*

Domain analysis revealed systematic variation in performance across clinical task types, with the magnitude of multimodal advantage varying according to the visual processing demands of each domain (Figure 4). Performance differences were largest in domains requiring visual pattern recognition (Diagnosis and Complication Management) and smallest in domains relying primarily on textual protocol knowledge (Treatment and Wound Subtype Knowledge).

#### Diagnosis Domain

The Diagnosis domain, comprising 7 questions addressing wound staging, classification, and etiology determination, demonstrated the most pronounced performance differences across groups. Human experts achieved 86% mean accuracy (6/7 correct, range 71%‐100%), MLLMs achieved 81% (5.7/7 correct, range 71%‐95%), and ChatGPT 5.0 achieved only 43% (3/7 correct). The Kruskal-Wallis test revealed highly significant group differences (*H*=11.24, df=2; *P*=.006). MLLMs outperformed ChatGPT 5.0 by 38 percentage points (*P*=.008), while humans outperformed ChatGPT 5.0 by 43 percentage points (*P*=.005). The human-multimodal difference was only 5 percentage points (*P*=.62, ns). This domain requires visual pattern recognition for pressure injury staging (Stage III vs IV differentiation), Wagner classification, venous versus arterial ulcer differentiation, and deep tissue pressure injury identification—tasks where verbal descriptions cannot adequately convey visual information necessary for accurate assessment.

#### Treatment Domain

The Treatment domain, comprising 6 questions addressing therapeutic interventions and management protocols, showed narrowed performance differences. Human experts achieved 88% (5.3/6 correct, range 67%‐100%), MLLMs achieved 73% (4.4/6 correct, range 67%‐83%), and ChatGPT 5.0 achieved 67% (4/6 correct). Group differences did not reach statistical significance (*H*=4.21, df=2; *P*=.06). The multimodal advantage over ChatGPT 5.0 was only 6 percentage points (*P*=.58, ns), substantially smaller than the 38-percentage-point advantage in Diagnosis. This domain tests knowledge of negative pressure wound therapy indications, compression therapy protocols, debridement selection, and antimicrobial agents—information extensively documented in clinical guidelines and accessible to both multimodal and unimodal models through text-based training.

#### Complication Management Domain

The Complication Management domain, comprising 6 questions addressing recognition and management of wound complications, demonstrated strong human superiority alongside significant multimodal advantage. Human experts achieved 92% (5.5/6 correct, range 83%‐100%), MLLMs achieved 72% (4.3/6 correct, range 50%‐83%), and ChatGPT 5.0 achieved 50% (3/6 correct). Group differences were significant (*H*=9.87, df=2; *P*=.01). Humans outperformed ChatGPT 5.0 by 42 percentage points (*P*=.003) and MLLMs by 20 percentage points (*P*=.048), while MLLMs outperformed ChatGPT 5.0 by 22 percentage points (*P*=.03). This domain requires integration of subtle visual findings—tissue color changes, exudate characteristics, periwound changes—with clinical context for infection-colonization differentiation, biofilm identification, and deterioration recognition. The substantial human advantage suggests experiential pattern recognition remains superior to current AI capabilities.

#### Wound Subtype Knowledge Domain

The Wound Subtype Knowledge domain, comprising 6 questions addressing classification systems and pathophysiology, demonstrated the smallest between-group differences and equivalent AI performance regardless of modality. Human experts achieved 83% (5/6 correct, range 67%‐100%), while both MLLMs and ChatGPT 5.0 achieved 67% (4/6 correct). Group differences were not significant (*H*=2.14, df=2; *P*=.11). The identical performance of MLLMs and ChatGPT 5.0 (67% vs 67%, difference=0%; *P*>.99) provides direct evidence that multimodal advantages are specifically attributable to visual processing capabilities. This domain tests factual knowledge about wound classification, healing trajectories, and management principles—information equally accessible to all AI systems through text-based training corpora.

### Response Time Analysis

Examination completion time differed markedly between human experts and AI models, with implications for clinical workflow integration. Human experts required a mean of 76.3 (SD 8.1) minutes to complete the 25-question examination (range 67‐85 min, median 76.5 min). Completion time varied inversely with expertise: the GS completed in 67 minutes (2.68 min/question), the WCN in 72 minutes (2.88 min/question), IM-1 in 81 minutes (3.24 min/question), and IM-2 in 85 minutes (3.40 min/question). The correlation between completion time and accuracy was strongly negative (*r*=−0.89, *P*=.006), indicating that specialized expertise enables both faster and more accurate performance.

All AI models generated responses essentially instantaneously, with total examination times under 1 minute regardless of model type. Med-PaLM 2 averaged 2.3 (SD 0.4) seconds per question (total 57.5 s), LLaVA-Med averaged 1.8 (SD 0.3) seconds (total 45 s), BioGPT averaged 1.1 (SD 0.2) seconds (total 27.5 s), and ChatGPT 5.0 averaged 0.9 (SD 0.2) seconds (total 22.5 s). The fastest human expert (GS, 67 min) was approximately 70 times slower than the slowest AI model (Med-PaLM 2, 57.5 s), representing a substantial efficiency advantage for AI systems. However, speed did not compensate for accuracy limitations in ChatGPT 5.0, which was both fastest and least accurate.

### Error Analysis

#### MLLM Errors

MLLMs collectively committed 16 errors across 75 question attempts (21.3% error rate). Error analysis revealed 3 predominant patterns. First, wound depth assessment errors accounted for 5 of 16 errors (31.3%), typically involving underestimation of tissue involvement (selecting Stage II when Stage III was correct or Stage III when Stage IV was correct). Two-dimensional photographs provide limited depth perception, making tissue layer differentiation challenging even with direct visual access. Second, infection versus colonization differentiation errors accounted for 4 of 16 errors (25%), with models demonstrating a conservative bias toward overestimating infection severity. Third, sequential clinical decision-making errors accounted for 3 of 16 errors (18.8%), occurring when correct answers required integrating multiple clinical steps (eg, Ankle-Brachial Index assessment before compression therapy). The remaining 4 (25%) errors were distributed across wound etiology differentiation, deep tissue injury recognition, and prognostic assessment.

#### ChatGPT 5.0 Errors

ChatGPT 5.0 committed 9 errors across 25 questions (36% error rate), with errors heavily concentrated in visually dependent domains: Diagnosis (4/7 incorrect, 57.1% error rate) and Complication Management (3/6 incorrect, 50% error rate) versus Treatment (1/6, 16.7%) and Subtype Knowledge (1/6, 16.7%). All Diagnosis errors involved failure to translate verbal descriptions into accurate visual assessments—consistently underestimating wound severity despite explicit textual descriptions of tissue involvement. On pressure injury staging, ChatGPT 5.0 selected Stage II when descriptions stated “visible subcutaneous fat” (Stage III criteria). On venous-arterial differentiation, ChatGPT 5.0 selected venous despite descriptions of “punched-out margins,” “pale wound bed,” and “absent pedal pulses”—classic arterial findings. This pattern confirms that sophisticated language understanding cannot compensate for the absence of visual processing in domains where image interpretation is essential. Errors were operationally classified as “information access limitations” when (1) the correct answer required information visually apparent in the wound photograph but verifiably absent in the verbal description, and (2) the model’s selected answer was consistent with the textual information provided. In contrast, “knowledge deficits” were classified when the correct answer could be derived from the available verbal description but the model failed to apply correct clinical reasoning. This classification was performed independently by 2 investigators, with discrepancies resolved by consensus. Specifically, to avoid circularity, the classification procedure involved a systematic cross-referencing step: for each ChatGPT 5.0 error, 2 independent investigators compared the correct answer’s informational requirements against the verbatim content of the standardized verbal description provided to the model. An error was classified as an “information access limitation” only when a specific visual feature necessary for the correct answer (eg, wound depth extending to bone, tissue color gradients, spatial distribution of necrosis) was confirmed to be absent from the written description through item-by-item verification against the 12-item template. Conversely, when the verbal description contained sufficient information to derive the correct answer (ie, all relevant clinical features were explicitly stated in the text), the error was classified as a “knowledge deficit.” Interrater agreement for this classification was substantial (Cohen κ=0.82).

#### Human Expert Errors

Human experts collectively committed 14 errors across 100 question attempts (14% error rate), with errors inversely correlated with specialized experience (*r*=−0.91; *P*=.004). The GS committed 1 error (4% error rate) on Ankle-Brachial Index interpretation—peripheral to surgical practice. The WCN committed 2 errors (8%) on Marjolin ulcer and Wagner classification—areas more central to surgical than nursing practice. IM-1 committed 5 errors (20%) and IM-2 committed 6 errors (24%) distributed across all domains. Specialized experts (GS+WCN) achieved a 6% error rate versus 22% for nonspecialists—a 3.7-fold difference confirming the protective value of domain-specific expertise. Notably, IM-2 and ChatGPT 5.0 shared 4 incorrect answers, suggesting that limited experience and text-only processing result in similar diagnostic limitations.

## Discussion

### Principal Findings

This study provides the first systematic comparison of MLLMs, unimodal large language models, and human clinical experts on a standardized wound care certification examination. Our findings demonstrate that while MLLMs show considerable promise in clinical wound assessment, they have not yet achieved parity with human expertise. Human clinical experts achieved the highest overall accuracy (86, SD 9.1%), followed by MLLMs (78.7, SD 12.2%), while the text-only ChatGPT 5.0 performed significantly below the certification threshold (64%). These results align with the emerging consensus that multimodal AI integration represents a critical advancement in medical imaging applications, yet substantial gaps remain before clinical deployment can be recommended.

The most striking finding was the pronounced multimodal advantage in visually dependent domains. MLLMs outperformed ChatGPT 5.0 by 38 percentage points in diagnosis (*P*=.006) and 22 points in complication management (*P*=.01), domains requiring direct image interpretation for wound staging, tissue assessment, and infection recognition. Conversely, no multimodal advantage was observed for wound subtype knowledge (both groups: 67%), which relies primarily on textual recall. This domain-specific pattern supports the theoretical framework proposed by Jung et al [17], who emphasized that MLLMs achieve their greatest utility when visual and textual information must be simultaneously integrated for clinical reasoning. Our findings empirically validate this framework in wound care, demonstrating that multimodal architectures provide meaningful advantages specifically in tasks requiring image comprehension.

Our results contribute to the growing body of evidence examining AI performance against human clinicians. Recent studies have shown that GPT-4 can achieve physician-level performance on medical board examinations, passing 4 of 5 specialty examinations in Israel, with scores exceeding the 65% threshold. Similarly, GPT-4 outperformed emergency department residents in diagnostic accuracy when provided with complete clinical information. However, these studies predominantly utilized text-based assessments. In contrast, our multimodal evaluation revealed that even the highest-performing MLLM (Med-PaLM 2, 92%) only matched the second-highest human expert, while 2 of 3 MLLMs failed to meet certification standards. This performance gap is consistent with Jin et al [18], who found that GPT-4V frequently presents flawed rationales despite achieving correct final answers, particularly in image comprehension tasks.

The superiority of human experts over ChatGPT 5.0 is statistically robust (*P*=.006, Cohen *d*=2.12). The GS with specialized wound care experience achieved 96% accuracy, outperforming all AI systems. However, for the MLLM-human comparison, the 7.3-percentage-point difference (*P*=.09) should be interpreted cautiously given the low statistical power (power=0.52); this finding suggests, but does not confirm, human superiority over MLLMs. Importantly, these 2 comparisons carry different levels of evidential strength: the human advantage over unimodal ChatGPT 5.0 is well established by our data, whereas the human advantage over MLLMs remains only suggestive due to insufficient statistical power, and an adequately powered equivalence or noninferiority trial would be required to draw definitive conclusions. This finding aligns with Kücking et al, who demonstrated that factors related to expertise—such as formal qualifications, deliberate practice, and diagnostic confidence—play a significant role in clinical judgment during wound care assessments [19]. Notably, the specialized human experts (GS, WCN) achieved 94% mean accuracy compared to 78% for nonspecialized physicians, suggesting that domain-specific training remains irreplaceable. The recent systematic review by Reifs Jiménez et al [20] similarly concluded that while AI systems excel at standardized pattern recognition, human clinicians demonstrate superior contextual reasoning and integration of atypical presentations.

From a clinical implementation perspective, our findings suggest that MLLMs may serve as valuable decision-support tools rather than autonomous diagnostic systems. The 67% pass rate among MLLMs (2/3 meeting the 70% threshold) indicates that top-tier models like Med-PaLM 2 can provide reliable second opinions in resource-limited settings. Grunhut and Nagarsheth [21] recently proposed that AI-powered wound assessment tools are best positioned for triage, preliminary documentation, and alerting clinicians to potential complications—functions that complement rather than replace expert judgment. Similarly, Barakat-Johnson et al [22] demonstrated that AI-assisted wound imaging improved standardization of assessments during the COVID-19 pandemic while maintaining physician oversight. Our data support this collaborative model: MLLMs excelled in standardized visual tasks while human experts demonstrated superior performance in nuanced clinical scenarios.

Recent advances in AI-powered wound assessment tools further contextualize our findings. A multicenter study by Swiss researchers (2025) demonstrated that deep learning-based wound segmentation achieved 92% DICE scores, comparable to expert annotations. However, tissue classification accuracy varied considerably across wound types, particularly for fibrin and necrosis—findings that parallel our observation of variable MLLM performance across clinical domains [23]. The emerging consensus from multiple 2025 systematic reviews emphasizes that while AI demonstrates high accuracy in standardized tasks, generalizability to diverse real-world presentations remains challenging. Russ et al [24] identified key barriers, including data quality heterogeneity, limited interpretability, and the need for standardized evaluation frameworks before widespread clinical adoption.

The efficiency differential between AI and human assessors merits attention. MLLMs completed the examination approximately 70 times faster than human experts, suggesting potential applications in high-volume screening contexts. Mohammed et al [25] reported similar findings, demonstrating that AI-powered wound measurement reduced assessment time by 75% compared to manual methods. This speed advantage becomes clinically meaningful in settings with limited specialist availability, where MLLMs could provide immediate preliminary assessments pending expert review. However, the inverse correlation between human completion time and accuracy (*r*=−0.89; *P*=.006) suggests that experienced clinicians process wound images more efficiently through pattern recognition developed over years of practice—a capability that current AI systems have not fully replicated.

Several limitations should be acknowledged. First, our sample size (n=8 participants) limits statistical power for subgroup analyses. Second, the standardized examination format may not fully capture the complexity of real-world wound assessment, where clinical history, patient interaction, and longitudinal observation inform decision-making. Third, rapid advancements in MLLM architectures mean that newer models may demonstrate improved performance. Fourth, the observed unimodal performance deficit likely represents a composite effect of (1) inherent text-only processing limitations and (2) potential information loss or observer-dependent bias during the human-mediated translation from clinical images to standardized verbal descriptions; the verbal description protocol was not independently validated, and the 12-item template (detailed in Multimedia Appendix 2) may not have captured all visually relevant features. Consequently, the observed performance differences between multimodal and unimodal models cannot be attributed solely to multimodality per se; they likely reflect a combination of direct image processing capability and the specific fidelity and completeness of the human-generated text descriptions. Future studies should incorporate independent validation of the verbal description protocol (eg, interrater reliability assessment) and consider alternative unimodal control conditions (such as automated image captioning) to disentangle these confounders. The recent development of region-grounded MLLMs with segmentation-aware spatial tokens, as described by Stefanelli et al [23], represents a promising direction for enhancing wound-specific AI capabilities. Future studies should evaluate these advanced architectures using larger, multicenter cohorts with prospective clinical validation.

### Conclusions

MLLMs demonstrate significant performance advantages over the state-of-the-art unimodal ChatGPT 5.0 on wound care certification examinations, with the multimodal advantage most pronounced in visually dependent domains such as Diagnosis and Complication Management. While human clinical experts with dedicated wound care experience maintain overall superiority, the point estimate of the top-performing MLLM (Med-PaLM 2, 92%) fell within the observed range of human scores; however, the underpowered MLLM-human comparison (power=0.52) and wide CIs preclude definitive conclusions regarding noninferiority or equivalence. These findings support the development of multimodal AI systems as clinical decision-support tools in wound care, while emphasizing the continued importance of human expertise and the need for adequately powered validation studies.

## Supplementary material

10.2196/88618 Multimedia Appendix 1 Graphical abstract illustrating the comparative performance of multimodal large language models, unimodal ChatGPT 5.0, and human clinical experts on a standardized 25-question wound care certification examination across four clinical domains (Diagnosis, Treatment, Complications, and Prevention).

10.2196/88618 Multimedia Appendix 2 Standardized 12-item verbal wound description template.

## Abbreviations

AI: artificial intelligence
COSMIN: Consensus-Based Standards for the Selection of Health Measurement Instruments
GS: general surgeon
IM-1: internal medicine physician 1
IM-2: internal medicine physician 2
MLLM: multimodal large language model
WCN: wound care nurse
