# Supplementary figures and images for: The Power of Multimodality in Multimodal Large Language Models, Unimodal ChatGPT 5.0, and Human Clinical Experts on a Wound Care Certification Examination: Cross-Sectional Comparative Study
Source: JMIR Form Res. 2026 Apr 27;10:e88618. doi: 10.2196/88618 (PMC13120536; doi:10.2196/88618)

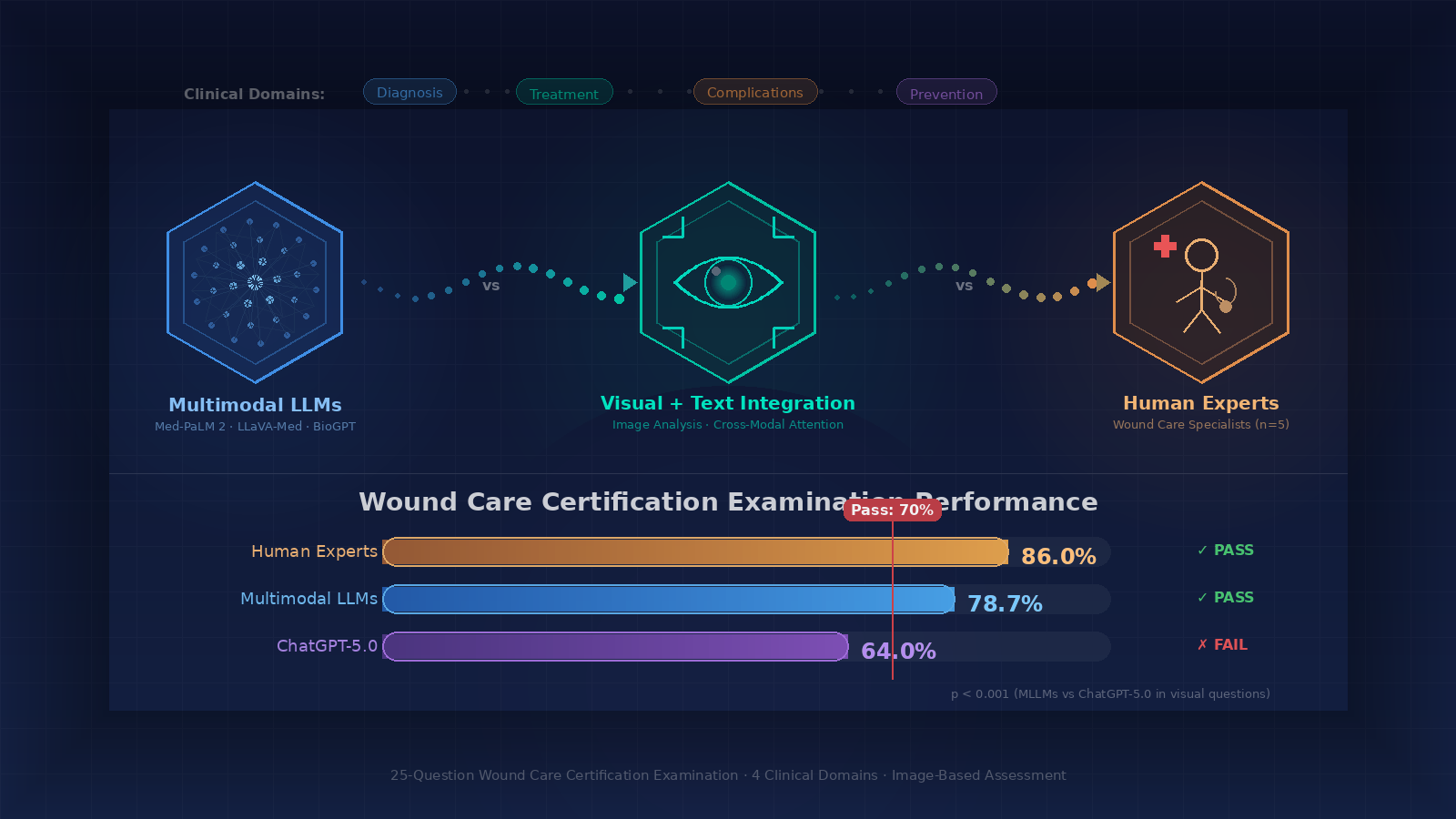

Supplement: Multimedia Appendix 1 [file formative-v10-e88618-s001.png]
