# Supplementary material for: The Power of Multimodality in Multimodal Large Language Models, Unimodal ChatGPT 5.0, and Human Clinical Experts on a Wound Care Certification Examination: Cross-Sectional Comparative Study
Source: JMIR Form Res. 2026 Apr 27;10:e88618. doi: 10.2196/88618 (PMC13120536; doi:10.2196/88618)
Supplement: Multimedia Appendix 2 [file formative-v10-e88618-s002.docx]

# Multimedia Appendix 2: Standardized 12-Item Verbal Wound Description Template

The following 12-item standardized template was used by a board-certified specialist (General Surgeon with 10 years of wound care experience) to generate verbal wound descriptions for the unimodal ChatGPT-5.0 condition. For each examination question containing a wound photograph, the specialist independently examined the clinical image and systematically documented findings according to each template item. The resulting verbal descriptions were used verbatim as input for ChatGPT-5.0 in place of direct image access. This protocol was not independently validated for inter-rater reliability; a single specialist prepared all descriptions to maintain internal consistency, which may have introduced observer-dependent bias.

**Template Items:**

1. Wound Location: Anatomical site, proximity to bony prominences or joints, laterality.

2. Wound Duration: Time since onset or first identification.

3. Tissue Loss Depth: Extent of tissue destruction through dermal layers (superficial, partial-thickness, full-thickness), structures visible or palpable in wound base (subcutaneous fat, fascia, muscle, tendon, bone).

4. Wound Bed Composition: Percentage and description of tissue types present (granulation, slough, necrotic/eschar, epithelializing tissue), color, texture, and moisture of each tissue type.

5. Exudate Characteristics: Type (serous, sanguineous, serosanguineous, purulent), amount (none, scant, small, moderate, large), color, odor, and consistency.

6. Wound Edge Characteristics: Edge definition (distinct, diffuse, rolled/epibole, undermined, attached, unattached), epithelialization status, and regularity.

7. Wound Size: Length × width × depth in centimeters.

8. Periwound Skin: Condition of surrounding tissue (intact, macerated, erythematous, indurated, excoriated, calloused, edematous), extent of changes from wound margin.

9. Infection Signs: Presence or absence of classic infection indicators (purulence, warmth, advancing erythema, edema, pain, foul odor), subtle biofilm indicators (shiny/slimy appearance, treatment-resistant wound), and systemic signs (fever, leukocytosis).

10. Tunneling and Undermining: Presence, direction (clock-face notation), and depth of any tunneling or undermining.

11. Pain Assessment: Pain level (numeric rating scale), temporal pattern (constant, intermittent, with dressing changes), and quality (sharp, dull, burning, throbbing).

12. Additional Visual Findings: Any other clinically relevant observations not captured above, including foreign bodies, exposed hardware, satellite lesions, skin grafts, surgical drains, or other notable features.
